# Dealing with a Latent Danger: Parents Communicating with Their Children about Smoking

**DOI:** 10.1155/2012/382075

**Published:** 2012-06-26

**Authors:** Sandra P. Small, Kaysi Eastlick Kushner, Anne Neufeld

**Affiliations:** ^1^School of Nursing, Memorial University of Newfoundland, St. John's, NL, Canada A1B 3V6; ^2^Faculty of Nursing, University of Alberta, 11405 87 Avenue, Edmonton, AB, Canada T6G 1C9

## Abstract

The purpose of this study was to understand parental approach to the topic of smoking with school-age preadolescent children. In-depth interviews were conducted with 38 parents and yielded a grounded theory that explains how parents communicated with their children about smoking. Parents perceived smoking to be a latent danger for their children. To deter smoking from occurring they verbally interacted with their children on the topic and took action by having a no-smoking rule. There were three interaction approaches, which differed by style and method of interaction. Most parents interacted by discussing smoking with their children. They intentionally took advantage of opportunities. Some interacted by telling their children about the health effects of smoking and their opposition to it. They responded on the spur-of-the-moment if their attention was drawn to the issue by external cues. A few interacted by acknowledging to their children the negative effects of smoking. They responded only when their children brought it up. The parents' intent for the no-smoking rule, which pertained mainly to their homes and vehicles, was to protect their children from second-hand smoke and limit exposure to smoking. The theory can be used by nurses to guide interventions with parents about youth smoking prevention.

## 1. Introduction

Tobacco use continues to be the leading cause of preventable morbidity and mortality in many countries and has been described as a global epidemic [1]. Smoking is most commonly tried and established in adolescence [2]. Tobacco dependence typically occurs in the early years of use, even at low levels of smoking [3, 4] and is considered a childhood condition [1]. Early age of smoking initiation is associated with heavy smoking over time [2]. Further, smoking during youth is associated with subsequent alcohol and illicit drug use during youth and for that reason it has been referred to as the gateway drug. The developing brain may be particularly susceptible to addiction, which makes primary prevention of smoking in youth all the more important [2, 5]. 

Despite a decline in some countries in recent years, youth smoking remains a major public health concern in many countries world-wide [6]. Within Canada cigarette smoking among adolescents aged 15 to 19 is at 14% [7]. Among younger children, ages 11 to 14 years, 22% have at least tried a cigarette [8]. Typically, smoking rates are based on cigarette use. Unfortunately, that tells only part of the story as many youths world-wide smoke other forms of tobacco, for example, little cigars and pipes [6, 8, 9]. 

Research efforts in the area of youth smoking primarily have focused on adolescents with the main emphasis being on identifying factors that influence them to smoke. Numerous studies have been carried out and a large number of correlates and predictors of the behavior have been identified, which may be broadly classified as social, psychological, personality, developmental, and genetic factors. One type of social influence that has been studied extensively is parental influence. Many parental characteristics and behaviors have been examined including smoking status, sociodemographic factors (e.g., education, income, and marital status), beliefs about smoking, attitude toward smoking, disciplinary measures for the child, rules restricting child exposure to smoking, and discussion with the child concerning smoking. 

However, an area that needs more research attention concerns parental communication with their children about the topic of smoking. In studies that have been carried out, generally the focus was narrow (e.g., whether discussion occurred or there were antismoking rules, or a particular aspect of communication such as frequency of discussion) and a comprehensive examination to gain an in-depth understanding was not taken. The studies largely were about communicating with adolescent or late preadolescent children and many were from the children's, not the parents', perspectives. Inconsistencies in findings make it difficult to draw conclusions about particulars of parental smoking-specific communication. No studies were found about parental smoking-specific communication with young school-age children. As well, a theory was not found that addresses parental communication with children about smoking.

Increasingly, it has been acknowledged that interventions to curb smoking should be broad, taking into account the varied influences [1, 10]. Yet, little has been done to engage parents in prevention efforts; for the most part, programs are not available to parents that would promote youth smoking prevention and media campaigns tend to be directed to youth themselves rather than to parents. Because adolescence is the key period for smoking initiation, to have an impact on prevention parents would need to take measures before the adolescent years. An important first step is to determine the approach parents typically take with their children before they become adolescents. The purpose of this study, therefore, was to understand from parents' perspectives their approach to the topic of smoking with their school-age pre-adolescent children. 

## 2. Method

We chose grounded theory method as it is suited to studying an area for which little is known or findings are unclear and gaining an in-depth understanding of a phenomenon through theory development [11, 12]. 

### 2.1. Sample

The study was approved by affiliated university ethics boards and written informed consent was obtained from the participants. The study took place in a city in eastern Canada. The participants were recruited through three means: (a) study information brochures sent home to parents through elementary schools; (b) study brochures and posters displayed in community centers, and (c) the snowball technique, whereby study participants identified other potential participants. Prospective participants were told that the purpose of the study was to learn about the approaches that parents take with their children about the topic of smoking. The purposive sample was comprised of 28 mothers and 10 fathers, including 6 mother-father pairs. The parents had at least one child 5 to 12 years of age (i.e., kindergarten to grade 6), with the majority (60%) having 2 or 3 children in that age range. There was about an equal number of boys and girls in the referent children. See Table 1 for a description of the sample characteristics.

### 2.2. Data Collection and Analysis

Consistent with grounded theory method, data collection and beginning analysis occurred concurrently. Interviews were carried out with the parents to encourage them to discuss their thoughts and feelings about youth smoking and smoking prevention and how they approach the topic with their children. Broad open-ended questions were employed such as “Would you tell me about your thoughts on children smoking?” “What are your thoughts on factors that influence children to smoke? “How has the topic of smoking come up?” “Can you think of a specific time when your child mentioned smoking or asked questions about it? Would you describe the situation for me?” “What do you find helpful to you (hinders you) in addressing the topic of smoking with your children?” “What are your thoughts on barriers to preventing smoking among children?” Parents' responses were probed for details. The interviews ranged in length from 30 to 60 minutes. Four parents who were interviewed early in the study were interviewed a second time to expand upon points in the first interviews. The interviews were held in private, and when both parents in a family participated in the study, they were interviewed separately. All interviews were conducted by the first author. They were digitally-recorded and then transcribed verbatim to form the text for analysis. After each interview, the interviewer recorded journal notes about her impressions of the interview and any questions that needed to be raised in future interviews, particular observations of the participant, thoughts about the data, and feelings the interview provoked personally. Those insights were used to guide subsequent data collection and inform analysis. 

**Figure 1:**
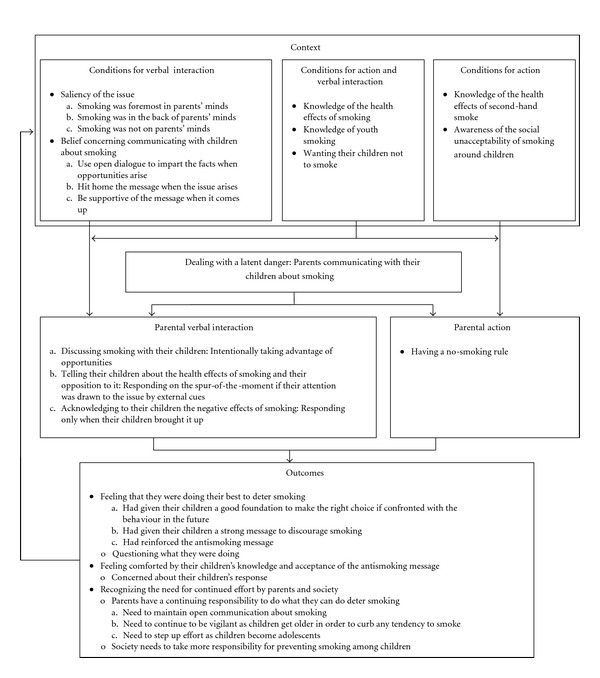
A theoretical model of the process that parents used in communicating with their children about smoking. Verbal interaction and action were influenced by conditions and resulted in outcomes for the parents. The outcomes fed back and contributed to the context for the parents' continuing action and interaction to deal with the latent danger. Note. The letters for conditions and outcomes correspond with the respective letters for the verbal interaction approaches and indicate variation according to the particular interaction approach.

The analysis was carried out primarily by the first author with team meetings to discuss findings and finalize the analysis. The procedure for constructing theory from the parent data was based on the approach of Strauss and Corbin [12] and involved coding and theoretical sampling. Coding consisted of three integrated steps. In the first step, open coding was used to identify concepts in the data and their properties and dimensions. Incidents were compared through constant comparison analysis for similarities and differences both within and across interviews. Incidents that were conceptually similar were grouped and labeled using *in vivo* codes, as possible, or substantively derived codes. In the second step, axial coding and the coding paradigm were used to link category with category and category with subcategory. This coding yielded the different types of interaction and the action the parents took with their children concerning smoking, the conditions that influenced their action and interaction, and the outcomes for them as a consequence of their action and interaction. In the third step, selective coding was used to integrate and refine categories and abstract a central category to form an explanatory whole. Throughout the coding process, memos were written to facilitate data analysis. Diagrams were created to help sort out relationships among the categories and culminated in Figure 1, the theoretical model. 

Theoretical sampling was used during data collection and analysis to achieve theoretical saturation. As concepts and relationships were identified in the data, those analytic leads were followed up with subsequent study participants. Previous interviews also were reviewed to consider whether there was any fit of new categories with previously identified categories. Theoretical sampling was conducted and data were collected until there was replication, no new information was arising during coding, and variation was accounted for. 

## 3. Results

The results represent a substantive theory that explains how parents communicated with their children about smoking. The central category *dealing with a latent danger: parents communicating with their children about smoking* represents the problem for the parents and their response to it (see Figure 1). The problem was that although their children were not smoking at that point in time, the possibility was there for them to begin in the future. As one parent said “you're dealing with a threat that's not immediate” (OA) (participants are identified by fictitious acronyms for multiword quotations). Although some parents thought of it as a more remote possibility because of their children's negative reaction to smoking, they had misgivings and a lingering uncertainty.

*I would be surprised. That would be my initial reaction to it because right now she has a real aversion to smoke.... I don't think that at this point... she would definitely not do [it]. Now like when she's a teenager it's going to be a different... you just don't know. (AM)*

Other parents thought that the possibility of their children beginning to smoke was more likely.

*Yes, I would be hurt but I wouldn't be surprised knowing that children are children and they're going to try different things…. You can't be like an ostrich and put your head in the sand…. You'll just be fooling yourself because then you're going to find out they're smoking, right, or found cigarettes in their pocket…[You] know because you did it yourself. (FU)*

This story illustrates the source of a mother's doubt.

*When she was about 6 or 7 she said, “When I get older I'm going to smoke” and I looked at her and said, “[Daughter], it's not good. It can do a lot of damage to your lungs.” I said, “It can give you cancer.” I said, “It's not a good habit to have.” “But,”she said, “daddy does smoking.” I said, “Yea, but daddy tells you everyday how he feels towards smoking. It's just a nasty habit.” And, he tells them that he don't like smoking, right. But, it's just a habit that.... And I said to her, “Why would you [say that]?” “I don't know,” she said, “mom.” She said, “Just wondering what it would be like if I smoked when I got older.” I'm like, “It's not a good habit.”... Now that she's 8, she says it is yucky. But, I mean, there's always a doubt in my mind. Is she going to smoke when she gets older? (CR)*

Hence, the meaning that parents applied to youth smoking relative to their children is that it is a latent danger. That meaning was shaped by their knowledge of the serious health effects of smoking and by their knowledge of youth smoking. Their response was to deter the behavior from materializing by communicating with their children, which involved verbally interacting with them on the subject and taking action in the form of having a no-smoking rule. Their verbal interaction and action produced outcomes for them in the form of feelings and thoughts. 

### 3.1. Parental Verbal Interaction

Parents verbally interacted with their children about smoking through using one of three approaches: (a) discussing smoking with their children by intentionally taking advantage of opportunities, (b) telling their children about the health effects of smoking and their opposition to it by responding on the spur-of-the-moment if their attention was drawn to the issue by external cues, or (c) acknowledging to their children the negative effects of smoking by responding only when their children brought it up (see Figure 1). Each approach is composed of interaction style, which refers to the manner in which the parents interacted with their children, and interaction method, which refers to what the parents did to interact with their children. The styles and associated methods reflect differences in the quality and extent of the parents' interaction. Each approach also is marked by underlying properties that reflect the purpose, timing, and intensity of the interaction and the character of the message conveyed. These reveal differences and similarities among approaches and within-category variation within approach. It is difficult to tell whether smoking status or any specific sociodemographic factor, including the sex of the referent children, was associated with a particular verbal interaction approach. However, there seemed to be a pattern of relatively fewer smoking parents, and more mothers, more parents who had a spouse or partner, and more parents with any of higher household income, education, and occupational status located in the category *discussing smoking with their children by intentionally taking advantage of opportunities* than in the other two categories. 

#### 3.1.1. Discussing Smoking with Their Children: Intentionally Taking Advantage of Opportunities

The majority of parents (22 of 38) interacted verbally with their children about smoking by discussing smoking with them, which reflects an open communication style. They encouraged their children to talk about smoking, engaged them in discussion, and participated with them in a two-way exchange of ideas. Their method was to take advantage of everyday ordinary opportunities. “It's utilizing whatever comes up at the time.... Every now and then something triggers it and we talk about it.” (AM) The discussions occurred “naturally” but were deliberate and “purposeful” nonetheless.“I look for a kind of teachable moment. I don't just say, okay, we're going to talk about smoking today and go from there.” (JV) Those parents had thought about it beforehand and had conscious intent to talk with their children about smoking.

**Purpose**

The purpose of the parents' interaction was to clarify or validate their children's understanding of smoking, give information about smoking, and reinforce the antismoking message.

**Timing**

Opportunities to discuss smoking occurred either from the parents noticing something themselves while with their children, such as seeing someone smoking, or from their children noticing something and making comments or asking questions about smoking.

*We just look for the opportunities. If there's an ad on TV, we'll pick up on that or if we're driving in the car, if there is an ad on the radio about not smoking then we'll, I'll pick up on that and just chat about it a bit. (EQ)*

In families where a parent smoked the topic came up often mainly because the child noticed and asked questions as to why the parent smoked or made negative comments about it. A mother, who smoked and whose husband also smoked, talked about her children's reaction, which gave her no choice but to discuss it. They would say things such as

*“You don't need to be smoking anyway. That stuff will kill you.”... and then they're talking about, “[I] can smell it off you, Mom. Go brush your teeth.”... The kids, they don't like it at all. They hate the fact that we smoke and they really get down on us.... Where I smoke I feel like I have to let the kids know what is going on with me. It is part of my life and it's part of their life so we have no other choice but to discuss it. (YK)*

**Intensity**

Parents started talking with their children before school-age. They believed that when children are old enough to grasp messages about health and “start asking questions about [smoking] then they're old enough to probably understand a little bit about it.” (JV) Some parents were sure to “take advantage of every opportunity” (IU) to convey an antismoking message. Other parents raised the topic more periodically, “not all the time but enough that it stays in their [children's] mind.” (AM) However, parents acknowledged that they needed to be careful to not “force” the issue or “harp” on it. It is important not to make the topic so common that it loses its effect, to have the “right balance” between raising it enough but not too much.

**Message**

The parents' emphasis in discussing smoking was on health effects including effects that were directly relevant to their children's personal situation such as effect on asthma and sports activity. They also tended to discuss other issues such as environmental tobacco smoke (ETS), the unacceptability of “pretend” smoking, and factors that influence people to smoke including, for current and some former smokers, their own addiction. Those parents thought that sharing their experience was a good teaching strategy. Other former smokers had not told their children they had smoked and were unsure whether they would for fear of it being a negative influence.

Although the parents gave an “honest” health message based on facts, some who formerly smoked or never smoked stressed the importance of using an “age-appropriate,” “progressive” approach. They took into account developmental level and tried to give a message that they thought the child would understand at his or her age. They used general messages about health and avoided talking specifically about cancer and death and giving graphic messages.

*I wouldn't introduce pictures or anything like you see sometimes on the back of cigarette packages.... Sometimes you'll see a picture of someone's mouth. It's been eaten away by cancer, or a set of lungs from a smoker or something.... I wouldn't want to shock them with horrible pictures. (JY)*

They thought that detailed and explicit messages about health consequences were more appropriate for children who were nearing or at adolescence; that is, once they are better able to understand disease, risk, probability, and long-term outcomes. Those parents were particularly mindful of what they said to their children if the other parent or a close relative such as a grandparent smoked as they didn't want to cause the children to become scared or worried. As the mother of young school-age children said

*I'm not going to talk to my children about that, especially with their father smoking. You don't really want to let them know that he might die from this.... They'd still get the message... it smells bad and it doesn't look very nice and it'll make you sick, even though their father is a smoker, being exposed to seeing him smoke. I still think they need that negative message... so I still give them everything negative that they can understand at their age about smoking. (IX)*

Other parents, regardless of smoking status, were less cautious in their approach. They always gave a strong, frank message to their children, even preschool children. They thought that children need and should not be protected from the blatant facts about smoking and that young children can understand about serious consequences. Where possible, those parents used real-life situations to show the serious health effects of smoking, for example, the illness or death of a grandparent. A mother conveyed that her father had died of lung cancer when her son was five years old and that she told him at the time why her father had died.

*We have been very up front in having discussions with him to let him know that poppy smoked for a long period of time.... and what smoking does and that smoking causes lung cancer and that the result of lung cancer is that in all likelihood you will die. And we have not kept that from him.... I want him to know that smoking does a lot of damage to your body, that ultimately it could kill you and I think that's the important message because I think that's the truth of it, and it's important for him and kids generally to know the truth about smoking. (EQ)*

Parents who had relatives who smoked and the parents who smoked themselves recognized that such messages can cause children to worry. However, they thought that, regardless of any emotional impact, it still was important for their children to know about the serious health effects. As one mother who smoked said “we discussed that smoking is not good for you and this [serious effects] is what happens. I've showed him the pictures on the cigarette packages and the nasty teeth and explained stuff to him.” (TI) For children who indicated that they might be troubled by the facts, parents tried to reassure them by explaining that while smoking is always harmful not every person who smokes ends up with serious disease or dies because of it and serious effects happen later in life. The parents who smoked tried to further comfort their children by indicating that they were fine and wanted to quit and would continue trying. A mother explained how she dealt with the situation when her son saw a television commercial of a smoker who had a tracheotomy and asked her

*“Like mommy, could that happen to you?” I couldn't say, no. When they ask you questions like that, what do you say cause you can't say no and I just said to him, “No, please God, mommy won't be smoking by then. Please God that won't happen to mommy.” Cause what can you say to them.... I just said, “No, hopefully mommy will never have to go through that.”(TI)*

#### 3.1.2. Telling Their Children about the Health Effects of Smoking and Their Opposition to It: Responding on the Spur-of-the-Moment If Their Attention Was Drawn to the Issue by External Cues

Some parents (9 of 38) interacted verbally with their children about smoking by telling them their thoughts about it, which reflects a directive style of communication. They did not engage their children in conversation about smoking as such. Their method was to comment about smoking if their “attention” was drawn to it by some smoking-specific external cue. For instance, a father said “if a commercial comes on TV about smoking and if they're [the children] doing something, I get their attention, “Look at that, look at that, pay attention”, right.” (AP) Their comments tended to be random and in the moment. “We don't have one specific time, one specific moment. It's just at that particular time and moment when it pops up.” (TH) Although parents' comments were goal-driven, that is, meant to deter smoking, their overall approach was not deliberately planned. It was spur-of-moment and is likened to a hit-or-miss approach.

**Purpose**

The purpose of the parents' interaction was to inform their children of the health effects of smoking and ensure they knew that the parents were opposed to it in an effort to persuade them not to smoke, “to make sure they do not get involved with it.” (NC)

**Timing**

The parents remarked about smoking when prompted by such cues as a question or comment about smoking from their children, exposure to smoking, and smoking-attributable illness in the family. As one father revealed, he had not said anything about smoking to his children before their grandfather had become ill with lung cancer and died because of it.

**Intensity**

The topic of smoking had first come up with their children before the children were school-age. Most parents had commented about smoking only occasionally over time and those parents tended to be moderate in their approach. Others varied in the frequency with which they “reiterated” their message about smoking, from occasionally to often, but they tended to be hardline in their approach. As one father said “I'm not going to sit there every day and tell them don't you smoke today.... But if the topic does come up, well I give [them] more than a mouthful.” (AP)

**Message**

The parents who had a moderate approach kept information about the health effects simple such as “it can make you sick.” (GV) Those parents thought that their children, who ranged from early to middle school-age, were too young to understand the serious health consequences and they would give a stronger message about the health effects when their children were older. Parents who were hardline in their approach told their children about the serious health consequences of smoking. They did not differentiate their message based on the child's age but believed that children should receive the strongest message regardless of age so “they'll listen and they'll remember.” (AP) They wanted their children to know the “real reality of it” (AP) and believed that fear was good for them. They used examples of family members, where possible.

*I had an aunt that died with lung cancer and I told them it had to do with smoking and I had an uncle that had to have his throat sliced on both sides and opened because of throat cancer and I told them that it all had to do with smoking.... And like your arteries are blocking and like that's what I explain to my 9 year old and she understands it. (NC)*

To send a strong message, those parents made firm, unequivocal statements such as “smoking kills.” A father said that he wanted his children to have the “message” that

*It'll kill them.... Not it'll make you sick, not it'll make you unpopular... just it'll kill you. You will die from this soon. I don't like the idea that you can say that someday this'll probably make you sick.... It [cancer] will kill you if you get it. This will give it to you. No sense of correlation. An absolute sense of causation. (OA)*

Parents who had smoked or who were currently smoking also had commented on their own smoking in an effort to reinforce the health message. 

Regardless of the strength of their health message, parents voiced their opposition to smoking by making sure their children knew that they were against the behavior or that they expected them not to smoke. The smoking parents realized they were not being good role models and wanted their children to get the message “do not do as I do, do as I say.” (GV)

*Well, basically, like he knows it's wrong. I know it's wrong.... Just because daddy does it, doesn't make it right. Just because daddy does it all the time, everyday whatever, you know, it's not right.... It's just the way I guess that they were raised. Since day one, it was put in their head that smoking is wrong even though I do it. Just because I do it doesn't make it right. It's wrong. (DS)*

Hard-line parents gave their children warnings that smoking would not be tolerated or told them of the punitive consequences they would get if ever caught smoking.

*I tell them, “You better not go smoking anyway cause I'll come get you. I'll find you.” So, if they goes having a smoke they're looking around the corner to see if I'm there cause I got that put in their head.… I just put it there and keep it there in a good way, you know, there's no harm, right. I tell them they'll get everything out of their room, all of their toys, the TVs, everything, gone.... And we always check their clothes. (AP)*

#### 3.1.3. Acknowledging to Their Children the Negative Effects of Smoking: Responding Only When Their Children Brought It Up

For a small group of parents (6 of 38), their verbal interaction with their children about smoking consisted of acknowledging to them the negative effects of the behavior. These parents had a nonassertive style of interacting with their children in that they did not raise the topic or enter into a conversation with them. As one mother of an 11-year-old daughter said “I just have not really had a conversation about that yet.” (HW) Rather, the parents simply confirmed the children's understanding of smoking. Their method was to comment only when their children brought it to their attention. For instance, one mother said that the topic had come up with her daughter only since grade 6 and it was the daughter who had raised it. Although these parents believed that in order to prevent smoking it is important for children to be informed, they did not take on an active role themselves. Their responses were routine rather than considered.

**Purpose**

Up to that point in their children's development, the parents had not given much consideration to smoking as an issue that needed their attention. However, they did not want their children to smoke, so their responses to them were to convey the message that smoking is not good for you or that it is harmful to health and that “no one should smoke.” (KZ)

**Timing**

The parents' interaction with their children about smoking was dependent on the children bringing it up. They simply responded to questions or comments the children made when they were provoked by such things as having done something in school about smoking or having seen antismoking signage or someone smoking. As one mother who was referring to her five year old said “I've never approached it.... He's had a few questions about what it is and so I've responded to his questions. I've never actually said anything just outright about it.” (LA)

**Intensity**

The parents had not initiated discussion with their children about smoking so there was no intensity on their part. However, some children had noticed and had asked about smoking before they were school-age, whereas others had not commented until they were older. Similarly, some of the children had raised the topic only occasionally; others had raised it often.

**Message**

The parents let their children know that they were correct about the negative attributes of smoking, but they did not offer extra detail or explanation about the behavior or explicit information about the health effects.

*I assure him that he's right, like, “Hey, you're right, you're not allowed smoking around here.”... Most of the times, after he points out that there's a no smoking sign, he'll say, “Smoking is bad for you” and I'm like, “You're right, smoking is very bad for you.”(KZ)*

The parents who smoked invariably had been confronted by their children about it in such ways as pointing out the discrepancy between what the parents were saying and doing and urging them to quit. “I try to tell them, when it comes up, that it's not good. It will make you sick. Of course they shoot back and say, “Well why do you smoke?”... and give me grief for smoking.” (WI) A mother said that her son had told her “that I should quit and he doesn't want me to smoke and it's bad for me and he wants me to be around to take care of him.” (QF) The parents tried to appease their children by indicating that they knew they should not smoke or suggesting that they would like to or intended to quit. “I put them off and say, “Daddy's going to quit soon. One of these days Daddy's going to throw them down.”... My famous escape is “soon.”... It's easy to brush it off and carry on to the next conversation.” (WI)

#### 3.1.4. Conditions for Parental Verbal Interaction

 Whether because of direct personal experience as a smoker or former smoker, knowledge as a result of having relatives or friends who smoked, or knowledge acquired more generally, parents knew that smoking causes serious illnesses and is a serious addiction and knew about youth smoking (see Figure 1). Many of the parents had family members or friends who had smoking-related illness or who had died as a consequence of such illnesses. Parents who formerly or currently smoked had experienced or were experiencing respiratory symptoms and had firsthand knowledge of addiction.

*I know what smoking does to you and how hard it is to quit. Like I'm after trying umpteen times.... I just cannot quit. I'm after trying the patch and the gum... but I just become so irritable that I actually find it hard to be a good mom when I don't smoke. But when I get out and have that cigarette, I come in and I can clean up my house. I can play with my children, read stories.... Once you get that craving it's just the worst thing in the world.... I just can't help myself. I just get the shakes and I just start crying and I just get really emotional and just got to have a cigarette. (YK)*

They especially knew how easy it is to start smoking and how quickly one becomes addicted. The parents' knowledge of the health consequences of smoking was the main reason for their verbal interaction. Because of the health effects the parents did not want their children to smoke. 

The parents had good knowledge of the nature of youth smoking and factors that influence children to smoke, which heightened their awareness of the vulnerability of children to smoking and gave them increased reason for their interaction. Although they thought that youth smoking was less common than when they were growing up, they believed that many youths still take up the behavior as they regularly saw them smoking. They knew that it more commonly occurs in adolescence but younger children also might try or even start smoking. Commenting about how young he was when he started smoking, a father said “I think I got caught smoking Camel cigarettes when I was 9 years old.” (AP) Some parents had seen smoking among preadolescents, even currently. Parents believed that children may begin to smoke for reasons such as exposure to other youths who smoke, role models who smoke (e.g., parents, siblings, and popular idols), and prosmoking messages in society (visibility of smoking and tobacco products) and relatively easy access to tobacco products. However, smoking by peers and family members generally was recognized as the most important. Many of the parents could relate personally to peer pressure because they had experienced it themselves when they were growing up. “It put you in a higher bracket like as in being cool around the school.” (XJ) Similarly, many of the parents who formerly or currently smoked could relate personally to the negative influence of family members, especially their own parents, who smoked. Some thought that their parents' smoking had been the “root” cause of their own smoking. Smoking parents acknowledged that their smoking was a negative influence for their own children.

*Growing up for me, I saw my parents smoke, figured it was okay, so I tried. Then I got hooked, been smoking ever since basically. But definitely parents play a humungous role in how their kids react and what their kids do. If they see their parents... smoking... obviously they're going to think it's okay and they're going to try it. If mom and dad can do it, why can't I, basically. (DS)*

Whereas their knowledge influenced the parents to verbally interact with their children about the topic of smoking, the saliency of the issue for them and their belief concerning communicating with children about smoking influenced the particular verbal interaction approach they took (see Figure 1). For some parents, their knowledge about the serious health consequences of smoking caused them strong emotions, such as deep concern or worry, sadness, and guilt, which kept smoking foremost in their minds or as one father said “top of mind.” (JY) Because smoking was so present in their consciousness or salient for them, when opportunities arose, and in an effort to deter the behavior, they intentionally took advantage and discussed the behavior with their children to ensure that they were well informed. The parents' emotions were evoked for any of several personal experiences: (a) being exposed to the health risks as a smoker or former smoker, regardless of whether there was any evidence of ill effects; (b) having a close family member who smoked and was at risk for illness, had serious illness, or had died from such illness; (c) having a child who had asthma, which could be worsened by smoking; and (d) having negative parental role modeling in the family because they or the other parent smoked.

*I think it makes me more desperate... to try to get that message across than it would if I wasn't a smoker cause I'd probably just tell them stuff. And it'd be like … that's nasty, blah, blah, blah. I think as a smoker, it's almost like I know if they grow up and they smoke I'm going to feel like I failed and I'm going to have guilt. So, I think like that's a big thing, is trying to avoid that whole thing by making sure they don't smoke. (TI)*

For other parents smoking was in the back of their minds rather than being foremost. Their response was not emotional but was matter-of-fact, a gut reaction that smoking is “horrible,” “disgusting,” and “atrocious by far … [so just] don't do it.” (DS) Those parents told their children about the health effects of smoking and their opposition to it if their attention was drawn to the issue by external cues. There also were parents for whom smoking was not on their minds. Although they did not condone smoking, they also did not respond emotionally to it. Their response was more neutral as reflected in the view that “I think everybody knows the cons of it, the health [effects].” (WI) Their approach was to acknowledge the negative effects of smoking by responding only when their children brought it up. 

 A similar pattern of variation in verbal interaction was noted for parental belief regarding communicating with children about smoking. Some parents believed it is important to use “open dialogue” to impart the facts when “opportunities” arise. Those parents discussed smoking with their children by intentionally taking advantage of opportunities. Their thoughts were that parents should be “honest... objective, non-punitive, and non-judgmental when discussing smoking.” (LX) Talking to children about smoking is about “equipping [them] to deal with things [rather than simply telling them] don't smoke. [It should not be] the Ten Commandments.” (RG) Open dialogue is the foundation for a positive relationship between children and their parents, increasing the chances that children will talk to their parents and accept the antismoking message in the long run. Parents believed that taking advantage of ordinary opportunities is a good strategy for initiating discussion with children about smoking and allows smoking education to be carried out in an ongoing manner throughout childhood.

Other parents believed it is important to “hit home” the message that smoking is “harmful” and “unacceptable” when the issue arises. They believed that smoking is an issue to which parents need to pay attention and address from time to time as well as on an as needed basis, that is, when the risk increases, such as with adolescence, or smoking actually materializes. Those parents told their children about the health effects of smoking and their opposition to it by responding on the spur-of-the-moment if their attention was drawn to the issue by external cues. 

Yet, other parents believed there was no need for them to do anything more at the time except be supportive of the antismoking message when it came up because their children already had received information about smoking through social sources, especially school. The approach of those parents was to acknowledge to their children the negative effects of smoking by responding only when their children brought it up. Parents of young school-age children thought that young children need only simple messages and their children had received those. They thought that detailed and explicit messaging is more appropriate for older children. Parents of older school-age children thought their children were very well informed about smoking. “It's already being talked about. What more do you do if it's after being talked about.” (HW) 

### 3.2. Parental Action

The main intent of the parents' no-smoking rule (see Figure 1) was to protect their children from ETS but they also wanted to limit exposure of their children to smoking behavior. The rule was consistent with and lent support to the message they conveyed through their verbal interaction that smoking is unhealthy. Although the strictness of the rule varied among parents from stringent to less stringent, there did not appear to be a pattern in stringency according to smoking status, sociodemographic characteristics, or particular verbal interaction approach. The parents who had a stringent rule held strong views against exposure to ETS and smoking. They were strongly opposed to smoking in public places that were visible and accessible to children, had a total ban on smoking in their homes and vehicles, and made a point of not exposing their children to ETS and smoking in places outside their homes, including the homes of relatives.

*We won't even go to like activities that the family has if people are going to be smoking and everybody knows that.... [Grandparents] go outside now, like, on account of the kids cause they know that I'm totally against it and I wouldn't bring them [the children] if I knew they were smoking in the house. I'm that against it. (BQ)*

They believed that a strict no-smoking rule demonstrates that it is not an “acceptable” behavior. The smoking parents always smoked outside and tried to do so inconspicuously so as to not draw their children's attention to it.

*First and foremost it's not allowed in my home. If I want to have one, like I said, snow, rain, whatever, I will go on outside and do my business.... I do go out by the door but I mean I don't announce and say, I'm going out to have a cigarette now. I kind of sneak out and do my thing and kind of sneak back in. I try to not let her even see me do it if I can. Like she knows that I do [smoke].... If somebody asked her if I did she wouldn't say no but can she say [I] see her do it all the time? She'd definitely have to say no there. (GV)*

The parents who had a less stringent rule tended not to require total avoidance of tobacco smoke and smoking. For instance, some had only partial restrictions on smoking in that they prohibited smoking in their homes and vehicles when their children were present but otherwise allowed it. Although parents who smoked did so outside when their children were home, they did not take extra precautions to conceal from their children what they were doing. They tended not to make an issue of environmental exposure beyond the societal measures that already were in place and tended not to be rigid about exposure in relatives' homes. Former and never-smokers in that group had a less stringent rule to accommodate a spouse or other relatives who smoked. 

#### 3.2.1. Conditions for Parental Action

 Parents, to one extent or another, knew that ETS can affect health. That knowledge was the main impetus for their no-smoking rule. In commenting on his rationale for smoking outside, a father said “it's bad enough that I'm polluting my lungs. Why would I want to pollute my child's.” (DS) Parents also knew that smoking in the presence of children had become “socially unacceptable” and not smoking around children was the societal expectation. Although more directly related to their verbal interaction, their knowledge of the health effects of smoking, their knowledge of youth smoking, and wanting their children not to smoke influenced them to have a no-smoking rule to limit exposure of their children to the behavior, hence, reducing what they thought was a risk factor for youth smoking (see Figure 1). 

### 3.3. Outcomes

Although there was variation that corresponded with the verbal interaction approach they had taken, parents felt that they were doing their “best” to deter smoking (see Figure 1). However, despite that feeling, a few parents, regardless of the approach they had taken with their children and their smoking status, questioned in their own minds what they were doing; if it was the most appropriate. They wondered about such things as whether their antismoking message was too strong or not strong enough, they talked about smoking too much or not enough, and they gave enough detail or not enough detail for the child's age.

*They are well versed in what I have chosen to give them.... But whether it's the right thing, I don't know. What is the appropriate thing to tell any child about smoking... I'm a parent, I'm doing the best I can and I have no idea if it's right or wrong. (OA)*

Similar to the other parents in this study, those parents had not sought or used any particular smoking prevention resources in their efforts to deter their children from smoking. Like the other parents, they were guided by their knowledge about smoking and belief concerning communicating with children about the behavior. However, they acknowledged that they could benefit from having more information on youth smoking, prevention strategies, and communication with children about smoking and thought that a resource they could use with their children would be helpful.

In addition to feeling they were doing their best, parents also were feeling comforted by their belief that their children had knowledge of and accepted the antismoking message (see Figure 1). At the very least, the children knew that smoking is unhealthy and can make people sick and some knew about the serious illnesses and that smoking can cause death. The children demonstrated acceptance of the antismoking message through various reactions. However, some held stronger antismoking views than did others. Those children not only were “receptive” to the message but had “internalized” it. They were quite knowledgeable about smoking and could “make a very strong case for not smoking” (ZL) based on the health facts, were ardently opposed to it, made negative comments when they saw someone smoking, went out of their way to avoid tobacco smoke, expressed concern about relatives who smoked and wanted to encourage them to quit or actually tried by telling them about the dangers of smoking, and demonstrated antismoking assertiveness with family members. They were tuned in to the issue perhaps even more so than were their parents.

*He tells all of us... stuff like, “You'll get cancer. You're going to get cancer.”... He'd read the cigarette packages and he'd read the labeling on it and he'd say... “Cigarettes cause lung cancer. Why are you smoking if it causes lung cancer? Why would you do that?” And he knew stuff. He'll say that to us, you know. “This is what's going to happen to your teeth. This is what's going to happen to your lungs.” So, I'm hoping that he remembers that when he gets a teenager and someone passes him a cigarette. (TI)*

Some of the parents of those children, although pleased that their children were antismoking, had concern about their children's response. All were parents whose approach was to discuss smoking with their children by intentionally taking advantage of opportunities. Their children were inclined to inappropriately tell others, even strangers, that they should not smoke or to think negatively of people who smoked such as they are “bad.” As a consequence the parents felt they had to be careful about the message they conveyed in order to temper their children's reaction and had to correct any unintended misperception about smokers.

*My little boy will get so worked up that I have to stop him from marching up to other people and telling them not to smoke.... That's one of the reasons why I don't want to come on as strong as I do because I don't want him to get up on a soapbox and start. (RG) *

Although feeling they were doing their best and feeling comforted by their children's knowledge and acceptance of the message, parents recognized the need for continued effort. They knew that because of the nature of youth smoking and influencing factors, especially at adolescence, smoking was possible for their children and at some level wondered whether they would stay smoke-free when they were older. “And generally I'm wondering if they just toe the line. “Yes, mommy I'll never smoke” and they might.” (PB) Hence, they thought that because of the continuing threat that might become more pronounced at adolescence, parents have an important continuing “responsibility” to do what they can to deter smoking. However, what they thought they would need to do varied with their overall approach (see Figure 1).

*Say from 10 years old to say 18, 19 years old, if you can save them [in] that period of time like when the peer pressure is there all that, [if] you can save them from that, I think you're pretty well in the clear then. I do, right. And that's your responsibility because from the age of 10 to 18 they're your responsibility anyway. So do what you can, I guess. (AP) *

Parents also thought that because they can do only so much society needs to take more responsibility for preventing smoking among children (see Figure 1) and that children might be more inclined to accept a message that is received through different sources. Although generally pleased with societal efforts in recent years to curb smoking, parents thought that regulations should be further strengthened to reduce access by children to tobacco and to reduce public exposure of children to the behavior. However, the area parents thought would produce the greatest impact is with respect to smoking prevention education. When they were growing up there was little emphasis on smoking prevention in society generally. For many, aside from perhaps being told or warned not to smoke, their own parents had not raised the subject or talked with them about it. Parents believed that a lack of education about the health consequences was a main cause of the high rate of smoking in the past. They thought that “education is the best tool” (ET) for prevention. They recognized that there had been more smoking prevention education in recent years, but many thought that it was not enough. Although some parents thought that schools had good smoking prevention education, many thought that little was being carried out, especially in the early grades. They thought that smoking should be covered early and often in the school curriculum.

*I think that school is really important. They need to hear the message in school as well and they need to hear it not just once a year. It needs to come up on a fairly regular basis as part of the health program or whatever and I think it needs to start in kindergarten and repeat the message regularly and loudly every year. (BN) *

Some wanted more done at the community level and identified children and parents as key targets. They thought there was little in the way of smoking prevention advertisements and that television advertisements against smoking were a particularly good way to get the message across to children. They suggested that antismoking messages be produced for young children, even preschool children, and conveyed through children's television programming. Although comfortable with what they were doing themselves, consistent with the parents who had questioned their own approach, some parents were of the view that there needs to be an ongoing prevention initiative to increase parent awareness of youth smoking, inform them about the important role they can play in smoking prevention, and guide their approach. They thought there are parents who do not address smoking with their children and suggested that smoking prevention education materials be readily available to parents through such venues as schools and health clinics. 

### 3.4. The Context for Parental Continuing Verbal Interaction and Action

The parents' feelings and thoughts as a consequence of their verbal interaction and action were not endpoints but dynamic internal processes. Although some parents were uncertain about the appropriateness of the verbal interaction approach they had taken with their children and some were concerned about their children's strong reaction to the antismoking message, in general, parents felt they were doing their best to deter smoking and felt comforted by their children's knowledge and acceptance of the message. However, they recognized the need for continued effort and thought that parents have an ongoing responsibility to deter the behavior. Those feelings and thoughts gave them reason to continue their effort and as such contributed to the ongoing context for their continuing interaction and action to deal with the latent danger (see Figure 1). 

### 3.5. A Negative Case

There was one parent in this study whose approach did not fit with the theory. He was a former smoker who had quit smoking before becoming a parent and his daughter was a late preadolescent. Similar to other parents in the study, this father had knowledge of smoking and factors that contribute to youth smoking and did not want his daughter to smoke. However, he had never raised the topic or discussed smoking with her or said anything at all about it to her as he thought there was no need to do so. He thought that his daughter had learned about smoking in school and had good knowledge of the health effects of smoking. Further, she demonstrated a negative attitude toward the behavior. Therefore, he believed she would never smoke; hence, not a latent danger, and he did not interact with her about it. For those reasons, the approach of that parent is considered a negative case. “I don't talk to her about it. She knows the dangers and that, right. I don't think she'll ever smoke…. Not the way she acts now like [about] people smoking and that…. I can't imagine her smoking.” (BC)

## 4. Discussion

Although they differed in what they had done, parents in this study had communicated in some manner with their children about smoking. No studies were found in the literature concerning younger children, but similar to the parents in this study, there is evidence that many parents at least raised the topic of smoking with their late preadolescent or adolescent children (e.g., [13–16]). It is difficult to tell from most studies how much parents talked with their children and the type and extent of content. However, similar to some of the parents in this study, it was noted in other studies that parents did not talk often about smoking with their adolescent children [17, 18]. Like many of the parents in this study, it seems that the main focus of any communication about smoking was on health effects, although expectations or warnings not to smoke, financial cost, and peer pressure were addressed in some cases [19–22].

The majority of parents interacted with their children by discussing smoking with them, which reflected an open style and they believed that communication with children should be an open dialogue. Their style fits with what has been characterized as good quality communication and which has such attributes as attentive, responsive, acceptant, open (back-and-forth), meaningful, honest, nonjudgmental, nonpunitive, and relaxed. That type of communication is effective for positive child outcomes. Communication that is characterized by such attributes as one-sidedness, superficial, strained, conflictual, controlling, judgmental, or punitive does not facilitate positive child outcomes [23–25]. The overall approach of the parents who discussed smoking with their children also closely matches recommendations by authorities in the field of smoking prevention [26–28]. This includes their open style, method of taking advantage of opportunities, deliberateness, early initiation of discussion, and comprehensive message addressing health and influencing factors. 

Two areas in which parents who discussed smoking with their children varied in their approach are with respect to age-appropriate messaging and discussion of their former smoking. Some parents took into account their children's developmental level and tried to give age-appropriate messages, whereas others gave a strong message about serious health effects irrespective of their children's age. It is recommended in the literature that parents take a developmental approach to discussing smoking with their children [27, 28]. However, there does not appear to be any hard-and-fast rule about what to discuss with children at particular ages. It is suggested that since children mature at different rates and since parents know their children best, they may have a better sense of what is appropriate at different ages for their own children. Similarly, some formerly smoking parents had talked with their children about their past addiction; whereas, others were uncertain as to whether they would. There does not appear to be a specific recommendation in the literature about whether parents who formerly smoked should raise and discuss with their children their past experience with smoking. However, it is argued that parents who smoke should talk with their children about their experience [27], which is consistent with what the smoking parents in this study had done. 

In addition to verbal interaction with their children, parents in this study had a no-smoking rule albeit, for some parents, their rule was not strict. It is well accepted that ETS is harmful to health [29] and that exposure to smoking is a risk factor for youth smoking because of modeling and because it engenders a perception of acceptability [30–32]. Consequently, and consistent with the measures of the parents in this study who had a stringent rule, it is recommended that homes and vehicles should be completely smoke-free and parents who smoke should not do so in the presence of their children [26, 27, 33]. Whereas in the past it commonly was the case that parents did not have any restrictions on smoking in their homes [34–36], consistent with the findings in this study, many parents now at least have partial restrictions with the majority having a total ban [8, 13, 33, 37, 38]. 

## 5. Implications for Practice, Theory, and Research

It is generally accepted that parents are a potentially powerful influence on children's decisions to smoke and have an important role to play in smoking prevention [27, 39]. Consistent with that view, parents in this study recognized the need for parental intervention to deter children from smoking. Although different in style and method of interaction, many parents had taken it upon themselves to address smoking with their children and all had a no-smoking rule. Parents across the three verbal interaction approaches thought that they had a continuing responsibility to do what they could to deter smoking as their children get older. The parents had knowledge about the health effects of smoking, the nature of youth smoking, and factors that influence youth to smoke that is consistent with what is known about smoking. However, although parents felt they were doing their best to discourage smoking, some wondered whether what they were doing was the most appropriate and they thought that they could benefit from having more information on the matter. Similarly, although parents were feeling comforted by their children's knowledge and acceptance of the antismoking message, some were concerned about their children's strong reaction. 

Nurses are encouraged to work with parents through an empowerment model whereby parents' strengths and efforts are acknowledged and fostered and they are supported to participate in smoking prevention social policy [40, 41]. Those whose approach is consistent with recommendations in the literature need to be encouraged to continue their interventions with their children and offered reassurance about their approach. Parents need to know that children might react strongly to messages about smoking and be offered guidance on how to address it. Those whose approach differs from recommendations should be offered guidance on how to address the topic with their children to build on and enhance their efforts. Appropriate educational resources to assist parents need to be made available to them. There is evidence to support such interventions. Parents have suggested that interventions for parents about alcohol, tobacco, and other drugs should focus on practical information concerning how to successfully talk with children, how to raise the topic, and what to talk about, rather than on factual information about specific drugs [42]. Interventions with parents that promoted their involvement in prevention efforts concerning smoking resulted in more discussion with their children [43–46]. As suggested by the parents in this study and endorsed by smoking prevention advocates in the field, youth smoking prevention requires a multifaceted approach which involves the efforts of parents, schools, and society at large [1, 10, 26, 47]. Some parents thought there needs to be more smoking prevention education for children both in school and at the larger community level. Parents also thought that regulations concerning access to tobacco and exposure to the behavior need to be strengthened. Nurses are encouraged to partner with parents to enable their active engagement in smoking prevention advocacy. Because they *want their children not to smoke*, parents could be a strong force for supportive public policy. 

The theory generated from this study is about parental communication with children who are younger than adolescence. Because adolescence is a high risk period for initiation of smoking, parents might have a different approach with their adolescent children than with younger children. Indeed, some parents in this study indicated that they would give more detail or a stronger message to older children or would need to change their approach (i.e., step up their effort as their children become adolescents). Hence, research needs to be carried out with parents of adolescent children to determine whether and how approaches to the topic of smoking change with adolescent children. That knowledge may then be used to extend the theory derived in this study or generate another substantive theory to explain the phenomenon for that age group. Smoking is one of a number of risk behaviors in which adolescents engage. Others include drinking alcohol, using illicit drugs, and having unsafe sex [9, 48]. There is a need for a formal theory that explains how parents address with their children risk behaviors in general in an effort to prevent them. 

There was one parent in this study whose approach did not fit the substantive theory. Although not invalidating the theory, that case draws attention to another approach parents might take with their children about smoking, *not addressing the topic of smoking at all*. All the parents in this study were self-selected for participation. Hence, it is conceivable that there are other parents whose approach aligns with that case. It also is conceivable that there are other parental approaches to the topic of smoking that were not identified by this study. For instance, there might be parents whose behaviour indicates approval of smoking. There is evidence in the literature that some parents engaged in prompting behaviors, such as asking their children to bring them cigarettes, which actually could facilitate their children towards smoking [49, 50]. In future studies on parental communication with children about smoking it is important to explore for other parental approaches that might exist. Such findings could be used to further develop this substantive theory. However, little is known about the effectiveness of parental communication for smoking prevention. Research to establish the effectiveness of parental approaches to the topic of smoking could further inform health promotion practice.

This study was about parental approaches to the topic of smoking from the perspective of parents. In studies of adolescent children, there is evidence they have different perceptions of their parents' communication than do their parents [14, 51–53]. Further, there is evidence of differences between mothers and fathers and adolescent girls and boys in perceptions of parent-child communication [54]. In future studies it would be important to examine children's perspectives about their parents' approaches to the topic of smoking and their receptivity to parental messages. Parenting children about smoking happens within the context of the family with interactions occurring among parents and children. Research using a family approach, involving parents and children, could lead to further understanding of the complexity involved. Observation as a source of data would provide important information about parent-child communication and would be useful to validating findings of this study. There was diversity among the parents in this study in terms of socio-demographic factors and smoking status, and there were boys and girls in the referent group of children. However, it was not possible to determine whether any such parent or child characteristics influenced parental approaches. Many of the parents had a high educational level and there were few fathers relative to mothers and few smoking parents relative to nonsmoking parents. There is a paucity of information in the literature on parent and child characteristics that influence parental smoking-specific communication. Research needs to be carried out to further examine those characteristics and to explore for other potential influences such as parenting styles. Findings could be used to elaborate the conditions component of the theory.

## 6. Conclusion

This theory contributes new knowledge about parents' communication with their children concerning smoking. Notwithstanding the need for more research in this area, the understanding gained from the theory can be used by nurses in their interventions with parents about youth smoking.

## Figures and Tables

**Table 1:** Parent characteristics.

Characteristics		*n* ^a^
Marital status	Single	10
	Spouse or partner	28

Household income^b^ (Canadian dollars)	Low (<$29,000)	12
	Middle ($30,000–$89,000)	13
	High (>$90,000)	12

Education	Less than high school	5
	High school graduate	3
	Some university or college	13
	University or college graduate	17

Occupation	Professional	12
	Services, sales	5
	Skilled trades	6
	Stay-at-home mother	10
	Unemployed, disabled, student	5

	Current smoker	9
	Mother	5
	Father	4
	Former smoker	17
Smoking status	Mother	11
	Father	6
	Never smoker	12
	Mother	12
	Father	0

Note. ^a^ *N* = 38.

^b^Missing data for one parent.
